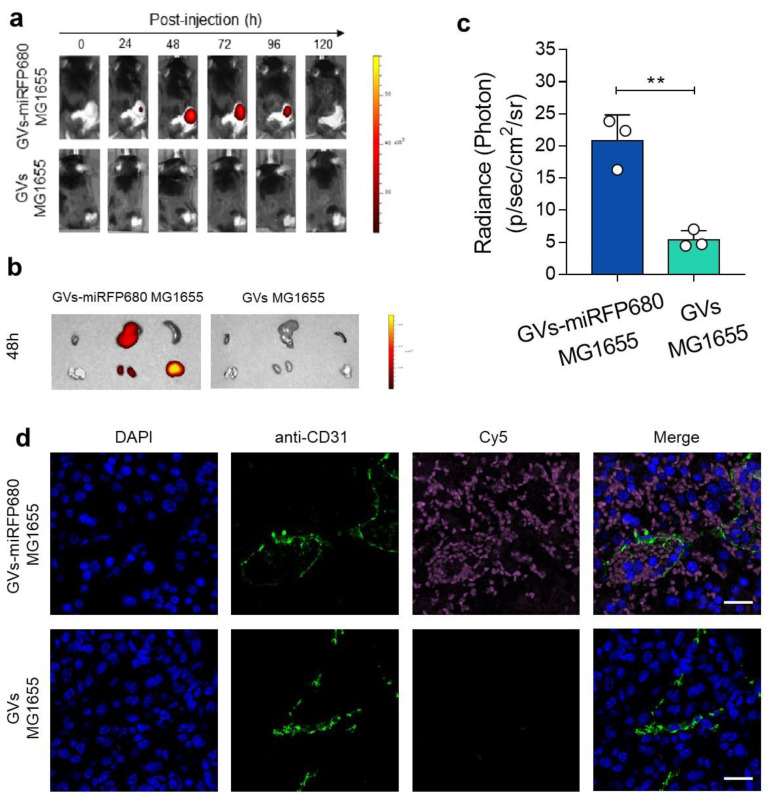# Correction: Zhang et al. Bimodal Imaging of Tumors via Genetically Engineered *Escherichia coli*. *Pharmaceutics* 2022, *14*, 1804

**DOI:** 10.3390/pharmaceutics15102516

**Published:** 2023-10-23

**Authors:** Linlin Zhang, Yuanyuan Wang, Dengjin Li, Liang Wang, Zhenzhou Li, Fei Yan

**Affiliations:** 1 Department of Ultrasound, The Second People’s Hospital of Shenzhen, The First Affiliated Hospital of Shenzhen University, Shenzhen 518061, China; lzz05432@163.com; 2 Shantou University Medical College, Shantou 515041, China; 3 Center for Cell and Gene Circuit Design, CAS Key Laboratory of Quantitative Engineering Biology, Shenzhen Institute of Synthetic Biology, Shenzhen Institutes of Advanced Technology, Chinese Academy of Sciences, Shenzhen 518055, China; yy.wang7@siat.ac.cn; 4 Center for Quantitative Synthetic Biology, CAS Key Laboratory of Quantitative Engineering Biology, Shenzhen Institute of Synthetic Biology, Shenzhen Institutes of Advanced Technology, Chinese Academy of Sciences, Shenzhen 518055, China; dj.li@siat.ac.cn; 5 Research Laboratory for Biomedical Optics and Molecular Imaging, CAS Key Laboratory of Health Informatics, Shenzhen Institute of Advanced Technology, Chinese Academy of Sciences, Shenzhen 518055, China; liang.wang1@siat.ac.cn

## Error in Figure

In the original publication [1], there was a mistake in Figure 6. In vivo tracking capability of the tumor-homing characteristic of GVs-miRFP680 MG1655. as published. Due to our carelessness in arranging the images in Figure 6a, the in vivo NIR fluorescent imaging data for the GV MG1655 group at the 0 and 24 h time points (the first two images in the second row) were mistakenly repeated. The corrected Figure 6a appears below. The authors apologize for any inconvenience caused and state that the scientific conclusions are unaffected.

**Figure:**